# Morphological Evolutions of Ni-Rich Phases in Al-Si Piston Alloys during 250–400 °C Thermal Exposure Processes

**DOI:** 10.3390/ma13225212

**Published:** 2020-11-18

**Authors:** Haowei An, Jiwei Geng, Zeyu Bian, Gen Liu, Mingliang Wang, Dong Chen, Haowei Wang

**Affiliations:** 1 State Key Laboratory of Metal Matrix Composites, Shanghai Jiao Tong University, Shanghai 200240, China; takonus@sjtu.edu.cn (H.A.); gengjiwei163@sjtu.edu.cn (J.G.); zybian@sjtu.edu.cn (Z.B.); g_liu2015@sjtu.edu.cn (G.L.); hwwang@sjtu.edu.cn (H.W.); 2 School of Materials Science & Engineering, Shanghai Jiao Tong University, Shanghai 200240, China; 3 Anhui Aluminium Matrix Composites Engineering Research Centre, Huaibei 235000, China

**Keywords:** Al-Si alloy, Ni-rich phases, thermal stability, morphological evolution, isochronous temperature curve

## Abstract

The thermal stability of the Al-Si alloys during the thermal exposure process from 250 °C to 400 °C was systematically investigated. The relationships between the morphological evolution and the mechanical changes of the alloys were determined through the Vickers hardness test and materials characterization method. Initially, the alloys exhibited similar thermal degradation behavior. For example, the exposure process of the alloy at 300 °C can be divided into two stages according to the changes of the alloy hardness and the matrix micro-hardness. In detail, the first stage (0–2 h) exhibited a severe reduction of the alloy hardness while the second stage showed a more leveled hardness during the following 98 h. There are three identified morphological characteristics of Ni-rich phases in the alloy. Furthermore, the differences in both composition and the micro-hardness between these Ni-rich phases were confirmed. The underlying relationships between the morphological transformation of the Ni-rich phases and hardness fluctuation in the alloy were correlated and elucidated. The observed alloy hardness increase when the exposure temperature was 400 °C was unexpected. This behavior was explained from the perspectives of both Ni-rich phases evolution and dispersoid formation.

## 1. Introduction

Al-Si alloys have been applied as heat-resistant Al alloys due to their excellent casting property and the interconnected eutectic Si structure [1,2,3]. The rigid Si network can bear more external load [4]. However, this eutectic Si phase tends to disintegrate and spheroidize when the alloy undergoes solution treatment or thermal exposure for a certain period of time, leading to a reduction of mechanical properties [5,6,7]. Practically, the Al-Si piston alloy has been developed by alloying strategy on the basis of eutectic Al-Si alloy [8]. Generally, the alloying elements with lower solubility and diffusivity in the Al matrix are selected. This method aims to introduce some new heat-resistant phases into matrix. The ideal heat-resistance phases should have a semi-continuous network built by the interconnected intermetallic compounds, which can grasp grains under applied load [2,9]. For instance, three types of Ni-rich phases (i.e., ε-Al_3_Ni, δ-Al_3_CuNi and γ-Al_7_Cu_4_Ni phases) are formed in Al-Si alloy when the transition metal elements (Cu, Ni) are added [10,11,12,13]. Both room temperature and elevated temperature tensile strengths of the Al-Si alloy are significantly improved due to the increasing volume fraction of the block ε-Al_3_Ni. Furthermore, the ε-Al3Ni phase can be regarded as an effective phase to improve the creep resistance of the Al-Si alloy [12]. The network δ-Al_3_CuNi and strip γ-Al_7_Cu_4_Ni are two main heat-resistance phases in the alloy [12]. Both phases can maintain their original morphologies even when exposed to elevated temperatures for a certain duration. This is caused by the low diffusion rate of Ni in the matrix [10,14]. Zuo et al. reported that the tensile strength of Al-Si alloys increased with the δ-Al_3_CuNi phase [14]. From the microscopic point of view, the decrease of the alloy hardness can be attributed to the interrelationships between the morphological interactions of the Ni-rich phase [15].

Both Cu and Mg are added to enhance the aging strengthening potential of Al-Si alloys. Heat treatment can be used to stimulate this latent capacity. For example, the formation of the semi-coherent precipitates and uniformly distribution of the precipitates in the matrix were stimulated by T6 treatment [16,17]. General accepted precipitation sequences in Al-Si alloy are shown as: supersaturated solid solution (SSS)→Mg/Si cluster→Guinier Preston zones (GP zones) zones→β″→β′→β [18], and (SSS)→Al/Cu cluster →GP zones →θ″→θ′→θ [18]. These precipitates (i.e., β′ and θ′) play an important role in improving the mechanical properties of the alloy at room temperature, due to their semi-coherent relationship with the matrix. However, the β′ and θ′ phases begin to coarsen when the temperatures exceed 170 °C and 200 °C, respectively. The semi-coherent precipitates (θ′ and β′) should completely transform into the incoherent stable phases (θ and β) when exposed for a certain period at these temperatures. This can be ascribed to the fast diffusion rates of Mg, Cu and Si in the matrix at elevated temperature. Since the pinning effect of the precipitates on grain boundaries and dislocations disappears, the strengthening effect on the matrix is reduced [19]. Moreover, the matrix softening should be more obvious when the external temperature exceeds 300 °C. Besides, Fe atom impurities are able to form a needle-shaped β-Al_5_FeSi phase in Al-Si alloys, which leads to stress concentration around it and reduces its elongation [11,20]. Mn has a high solubility and low diffusion rate in the matrix at high temperature [21]. Mn addition mainly improves the mechanical properties of the alloy from two aspects. Firstly, Mn can stimulate the transformation of Fe-containing phase from needle-like β-Al_5_FeSi to Chinese script-like α-Al_15_(Fe, Mn)_3_Si_2_ during the solidification process [22,23]. Secondly, a small amount of Mn can form the coherent dispersoids in the matrix at high temperature. This dispersoid strengthening effect can effectively improve the thermal stability of Al-Si alloys [24].

In the present work, the evolutions of the alloy hardness and the morphological evolution of the Ni-rich phases were firstly studied during a 300 °C exposure process. Afterwards, a morphologically quantitative method was established to distinguish the different morphologies of Ni-rich phases during the exposure process. The effects of the exposure temperature (250 °C to 400 °C) on the hardness evolution of the alloy were explored in detail. The variation process of second phases and the dispersoid in the matrix were also discussed.

## 2. Experimental Procedures

### 2.1. Materials Preparation

The eutectic Al-Si piston alloy was prepared from high purity Al (99.99%, all compositions used in this work are in wt.% unless specified otherwise), pure Mg, Al-20Si, Al-50Cu, Al-10Ni, Al-10Mn master alloys. These raw materials were melted in a clean iron crucible placed in an electric resistance furnace. The crucible was coated with boron nitride paint. First, the pure Al, pure Mg and other master alloys were melted in the crucible at ~750 °C. Then, the melt was refined at 760 °C for 10 min. In the next step, the melt was degassed in a vacuum furnace for 10 min. Finally, the melt was poured at 740 °C by gravity casting into an iron mold, which was preheated to 200 °C.

The composition of the as-cast alloy was tested by the inductively coupled plasma-atomic emission spectrometry (ICP-AES, ICP7600, American Thermo Company, Schaumburg, IL, USA). According to Table 1, the test result showed that the ICP value was in good consistence with its nominal composition, which confirms the effective control of the casting process.

The T6 treatment process includes two parts: first solution treatment and then artificial aging, which is suitable for products that are not cold-processed after solution treatment [25]. It is usually employed to improve the mechanical properties of heat treatable Al alloys, including Al-Si series and Al-Cu series alloys [26]. Consequently, these Al-Si alloys were heat treated with the T6 method. In detail, the as-cast alloy was solution treated at 480 °C for 4 h, and subsequently quenched into cold water. Afterwards, the alloys were instantly aged at four aging temperatures (160, 180, 200 and 235) °C with certain durations to achieve their peak-aged (PA) states. Generally, these Al-Si alloys at their PA states were labeled as T6 (160 °C), T6 (180 °C), T6 (200 °C), T6 (235 °C) alloys, accordingly.

### 2.2. Microstructure Characterization

The phase analysis of the Al-Si alloy was performed by X-ray diffraction (XRD, D8 ADVANCE Da Vinci, Germany Bruker manufacturer, Karlsruhe, Germany) from 20° to 50° at a rate of 2°/min. The morphological features were characterized by scanning electron microscopy (SEM, TASCAN MAIA3, Tascan, Brno, Czech Republic). The phase composition analysis was detected by an SEM system equipped with an energy dispersive X-ray spectroscopy detector (EDS, Tascan). A transmission microscope (TEM, JEM-2100F, Japan Electronics Corporation, Tokyo, Japan) operating at 200 kV was used for detailed microstructure observations. The precipitate compositions were analyzed by the EDS detector attached to the TEM equipment.

The TEM samples were thinned to ~80 μm by a mechanical method and then punched to obtain a sample with a 3 mm diameter. Then a dimple grinder (DG 657, American GATAN Company, Pleasanton, CA, USA) was used to reduce the thickness in the middle of the sample to ~50 μm. Finally, a precision ion thinner (PIPS, PIPS 695, American GATAN Company) was used to make an ideal thin area in the middle of the samples for TEM observation.

The size and distribution of these precipitates and second phases were obtained from TEM and SEM images using Image Pro Plus software (6.0 version, Media Cybernetics, Rockville, MD, USA). At least five photos of different areas were taken to estimate the average size of feature phases of the alloys exposed to various heat treatments.

### 2.3. Mechanical Properties

Thermal exposure experiments were employed to evaluate the thermal stability of the alloy under different heat treated conditions [21]. According to the service temperatures at different parts of the piston [24], four exposure temperatures (i.e., 250/300/350/400 °C) have been set to mimic and study the mechanical property evolution of different parts for the piston during practical conditions. The Vickers hardness (HV) can act as the indicator of mechanical property of these Al-Si alloys during the thermal exposure process [27].

The HV measurement was performed on a hardness test machine (Carat 930, EZ-mat, Denmark) with a load of 10 kg and dwell for 15 s. At least five points were tested, and the average value was derived for each sample. Both final hardness (FH, the hardness of the alloy after 100 h exposure) and hardness retention ratio (HRR, the ration of FH divided by the PA hardness) were used to evaluate the alloy’s thermal stability. On the other hand, the micro-hardness for a local area (i.e., Al matrix [28] or the micro-sized Ni-rich phases [29]) was performed with a load of 15 g and dwell for 15 s using the same test facility. In this case, at least 15 points were tested [30]. This hardness test method is similar to the nano-indentation, which can directly reflect the difference in mechanical properties of the alloy at local positions. For example, it is universally accepted that the precipitate is main factor to strengthen the alloy matrix [31]. Therefore, the micro-hardness of the matrix can be used to reflect the evolution of strengthening effect induced by the precipitate during the thermal exposure process.

## 3. Results

### 3.1. Microstructures of As-Cast of Al-Si Piston Alloy

**Figure 1:**
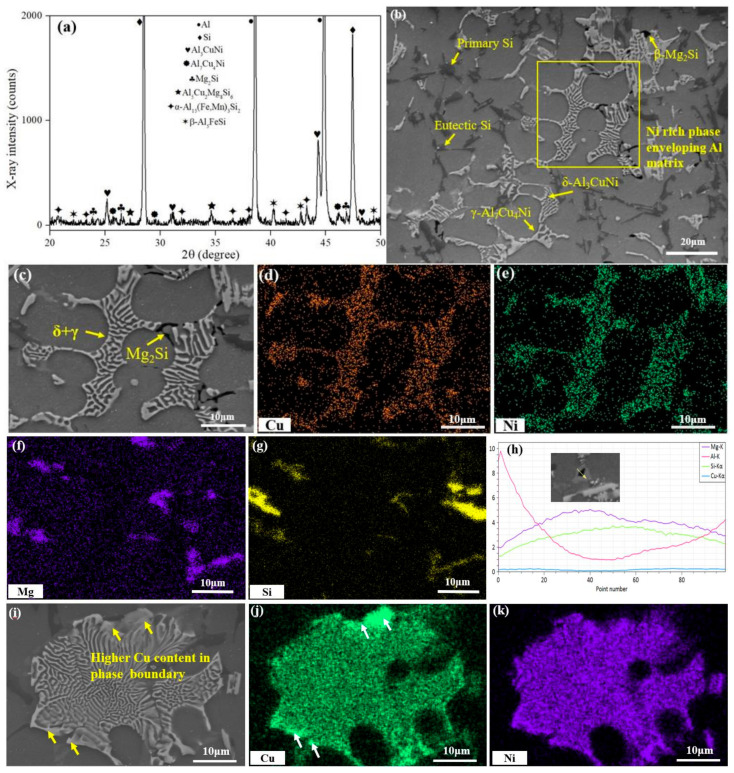
(**a**) XRD spectrum, (**b**) Typical microstructure, and (**c**) Local enlarged view of as-cast Al-Si alloy; (**d**) Cu distribution mapping of (**c**); (**e**) Ni distribution mapping of (**c**); (**f**) Mg distribution mapping of (**c**); (**g**) Si distribution mapping of (**c**); (**h**) Line scan results of Mg_2_Si phase; (**i**) Typical (δ + γ) peritectic structure; (**j**) Cu distribution in peritectic structure; (**k**) Ni distribution in peritectic structure.

As seen in Figure 1a, the as-cast Al-Si alloy is primarily composed of α-Al, Si phases, δ-Al_3_CuNi, γ-Al_7_Cu_4_Ni, Mg_2_Si, Q-Al_5_Cu_2_Mg_8_Si_6_, β-Al_5_FiSi and α-Al_15_(Fe, Mn)_3_Si_2_ based on the identification for its XRD spectrum. The general morphological details of the second phases in the as-cast alloy are shown in Figure 1b. In detail, the gray continuous phase is Al matrix and the dark grey part is Si phase with two categories (i.e., bulk primary Si phase and acicular eutectic Si phase). As revealed by [32], the strength of as-cast Al-Si alloy is governed by rigid eutectic Si phase due to its three dimensional network structure with the higher Young’s modulus embedded in ductile α-Al phase [32]. 

This situation is similar to fiber-reinforced composite materials. Overall, the primary Si phase is often regarded as the stress concentration point, which can evolve into a crack source when the applied load exceeds the load carrying capacity. This can be attributed to the larger modulus mismatch between Si phase and Al matrix [33].

A local enlarged view for the second phases in as-cast Al-Si alloy (Figure 1c), and the corresponding element mapping images (Figure 1d–g) are also exhibited. The bright grey part is the Ni-rich phases, which shows the network-like structure of short rod assemblies. This microstructure should be composed of δ-Al_3_CuNi and γ-Al_7_Cu_4_Ni as suggested by Yang et al. [10], where the γ-Al_7_Cu_4_Ni phase is transformed from the δ-Al_3_CuNi phase by the peritectic reaction:

(1) $${L+\delta \text{-}\mathrm{Al}}_{3}\mathrm{CuNi}\to {\alpha \text{-}\mathrm{Al}+\mathrm{Si}+\gamma \text{-}\mathrm{Al}}_{7}{\mathrm{Cu}}_{4}\mathrm{Ni}$$

The γ-Al_7_Cu_4_Ni phase is mainly distributed at the edge of network structure, which shows the coarser shape than δ-Al_3_CuNi phase [9]. Comparably, δ-Al_3_CuNi phase is generally distributed in the center of network structure. This complex peritectic structure can be confirmed by mapping results (Figure 1i). The phase with the higher Cu concentration is displayed at the edge, and the phase with lower Cu concentration is in the middle of the network structure. Normally, the Ni-rich phases are the key heat-resistant phase for the Al-Si alloy [13], which can be explained by two aspects. First, the Ni-rich phases can fix the alloy grains under the applied load or at higher service temperature [34]. Furthermore, the network-like structure can be kept even at higher temperature because of the lower diffusion rate of Ni in the matrix [28].

From the element mapping (Figure 1f) and line scanning results (Figure 1h), the strip shape can be determined as Mg_2_Si phase. This phase is usually dissolved into the matrix by solution treatment, and the coherent β″ or semi-coherent β′ phase should precipitate during the following aging process. However, both β″ and β′ precipitates are inclined to coarsen and lost their pinning effects on dislocations when the service temperature exceeds 170 °C [17]. Therefore, the Mg_2_Si phase has inferior contribution to the high temperature property of the Al-Si alloy.

### 3.2. Heat Treated Al-Si Alloys with Relative Thermal Stabilities

**Figure 2:**
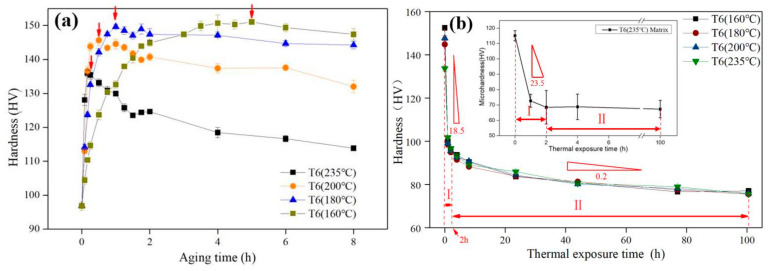
(**a**) T6 aging curves with four aging temperatures (160–235 °C); (**b**) Thermal exposure curves of four T6 Al-Si alloys to 300 °C; Insert: the matrix micro-hardness evolution of T6 (235 °C) Al-Si alloy.

In order to tune the mechanical properties of the alloy, the as-cast Al-Si alloy has been heat- treated using the T6 scheme. Herein, four aging temperatures (i.e., 160/180/200/235 °C) are adopted. In the aging curves (Figure 2a), there are several points that merit emphasizing. In the first place, the time for the Al-Si alloy to reach the PA state is shortened by a higher aging temperature. For instance, it takes 5 h to reach the PA state when the aging temperature is 160 °C. In contrast, it only takes 10 min to reach the PA state when the aging temperature is 235 °C. This is because the diffusion rate of alloying atom is increasing with the elevated aging temperature according to the Fick’s first law [35]. The formation and coarsening rate to critical size of the precipitate are accelerated at elevated temperature [36]. Secondly, the alloy hardness raises first (under-aged state), and then decreases (over-aged state) during the aging process. The hardness increment is attribute to the formation of nano-sized θ′ precipitates [37]. Clearly, the higher aging temperature has corresponded to the more obvious hardness reduction after the PA point at the over-aged stage (Figure 2a). Finally, the hardness values for the PA state are decreased with the elevated aging temperatures in the Al-Si alloy (Figure 1a and Table 2). It can be attributed to the larger critical size of the precipitate in the PA state at a higher aging temperature. In light of Orowan mechanism, the ability of the precipitate to hinder the movement of dislocations decreases as its size increases [38].

Figure 2b displays the hardness variations of four PA Al-Si alloys exposed to 300 °C for 100 h duration. Taking T6 (235 °C) as an example, this process can be divided into two stages based on the changes in HV values: (1) Stage I (0–2 h): the alloy shows 39.0 HV reduction with a larger softening rate (18.5 HV/h); (2) Stage II (2–100 h): the hardness has decreased at a slower rate (0.2 HV/h) and maintained the stable level with a smaller reduction of 19.6 HV. Comparatively, the micro-hardness on the Al matrix has been analyzed using the similar way, as shown in the insert of Figure 2b.

In the first stage (the first 2 h), the micro-hardness of the matrix decreases dramatically by 47.1 HV (with a decrease rate of 23.6 HV/h). Later, the micro-hardness reduction of the matrix is only 0.8 HV in the subsequent 98 h. Therefore, the hardness reduction of the PA alloy can be largely accounted for the weakening of the matrix in the first stage of the exposure process at 300 °C. Substantially, the sharp decrease of the hardness or the micro-hardness can be attributed to the rapid coarsening of precipitate [6,39]. Therefore, this process is closely related to the precipitate evolution [31].

In the second stage of the exposure process at 300 °C, the hardness of the PA alloy decreases slightly, and the reduction of the matrix macro-hardness is substantially minimized. As reported by Gaber et al., the precipitates have completely lost their original appearance and reached their thermodynamic stable state in this stage [40]. Therefore, the hardness behavior for the second stage should be related to the evolution of the second phase instead of the precipitate in the alloy matrix.

In Table 2, the hardness differences among these four PA alloys are quantitatively small enough to be negligible from the 2 h timepoint on. It is thus suggested that the aging temperatures of the T6 method has little effect on the final hardness of the Al-Si alloy experiencing long-term thermal exposure. However, it is worth noting that the Al-Si alloy at T6 (235 °C) state has the largest hardness retention ratio among the four PA alloys. It has thus indicated that the T6 (235 °C) treatment should include the least hardness degrade in the Al-Si alloy, which should be benefited to the piston at the practical service condition. Consequently, the PA alloy at T6 (235 °C) state has been thoroughly characterized and discussed in the following part.

### 3.3. Basic Morphological Features T6 (235 °C) Al-Si Alloys

The hardness of the as-cast Al-Si alloy was significantly improved after the T6 heat treatment. Then, it is necessary to compare the morphological evolution of the alloy before and after the T6 (235 °C) treatment. The XRD pattern and SEM microstructure of T6 (235 °C) Al-Si alloy are presented in Figure 2a,b. Compared with the as-cast alloy (Figure 1a,b), the main second phases are rarely changed. The X-ray diffraction pattern shows that α-Al, Si phases, δ-Al_3_CuNi, γ-Al_7_Cu_4_Ni, Mg_2_Si, Q-Al_5_Cu_2_Mg_8_Si_6_, β-Al_5_FeSi and α-Al_15_(Fe, Mn)_3_Si_2_ are all present in the T6 (235 °C) Al-Si alloy. However, there are some morphological changes for second phases induced by the T6 (235 °C) treatment. For examples, the network structure of eutectic Si phase has been disconnected and spheroidized after the T6 treatment. This leads to a decline in the load-bearing capacity of the eutectic Si phase [7]. Furthermore, the primary Si phase has more rounded edges by T6 treatment, which reduces the occurrence rate of stress concentration under the external load condition [41]. These morphological changes can be caused by the fast diffusion rate of Si atom in Al matrix at high temperatures. This morphological evolution is also reported by the recent references [4,42].

**Figure 3:**
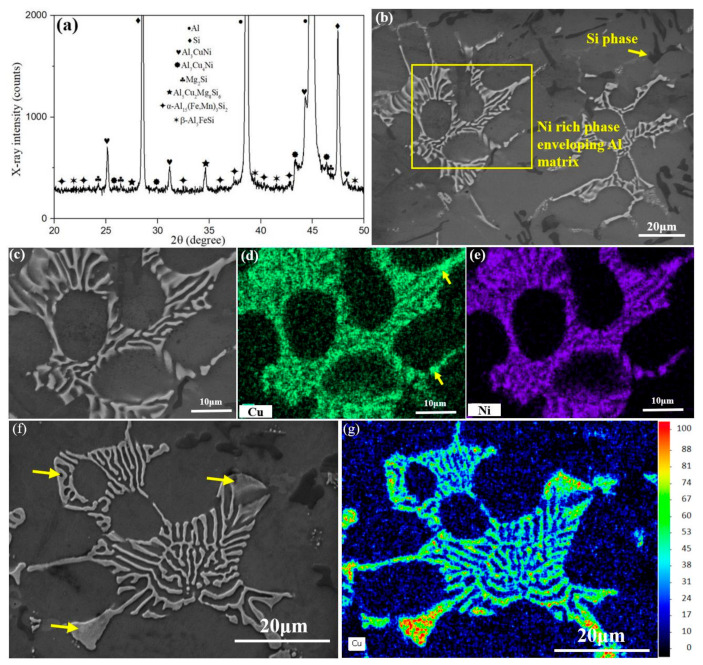
(**a**) XRD spectrum, (**b**) Typical microstructure, (**c**) Local enlarged view of T6 (235 °C) Al-Si alloy; (**d**) Cu distribution mapping of (**c**); (**e**) Ni distribution mapping of (**c**); (**f**,**g**) (δ + γ) peritectic structure and corresponding Cu distribution.

The local enlarged view for the second phases in the T6 (235 °C) Al-Si alloy (Figure 3c), and the corresponding element mapping images (Figure 3d,e) are presented. As pointed by yellow arrow and the red color part in Figure 3f, the Cu content at the edge region of Ni-rich phases is higher than that in the middle part. These uneven Cu distributions in the Ni-rich phases are similar to the previous results in Figure 1j, which should be (δ + γ) phases in the peritectic structure. These Ni-rich phases have good thermal stability, which can still maintain its original network structure after the T6 (235 °C) treatment (i.e., 480 °C for 4 h + 235 °C for 10 min). Generally, the Ni-rich phases are closely related to the thermal stability of the alloy, which should be discussed in subsequent contents.

## 4. Discussion

Notably, the T6 (235 °C) Al-Si alloy has the largest hardness retention ratio among four PA alloys. The 300 °C thermal exposure process of the T6 (235 °C) alloy can be divided into two stages: (1) the first stage refers to the first 2 h of exposure process, in which there is a sharp hardness reduction. This stage is closely linked with the evolution of precipitates in the matrix; (2) The second stage refers to the following 98 h with a smaller decrease in the hardness, which should be closely related to the evolution of second phases. Since the Ni-rich phases are considered as the key heat-resistant phase at elevated temperature, its morphological dependence on the hardness fluctuation for the Al-Si alloy during thermal exposure process should be specifically concerned. The effect of Ni-rich phases on the alloy thermal stability should be discussed hereafter from the perspective of microstructure evolution and isochronous temperature curves.

### 4.1. Morphological Evolution of Ni-Rich Phases during 300 °C Thermal Exposure Process

**Figure 4:**
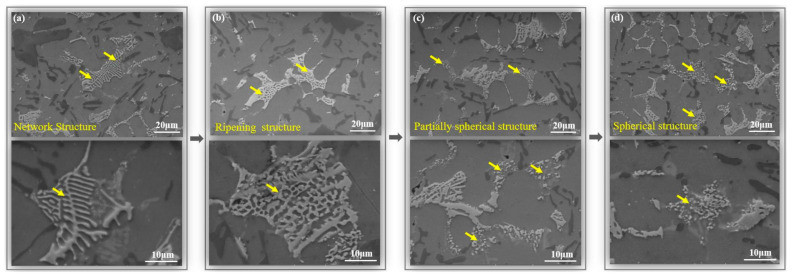
Typical morphological evolution of Ni-rich phases during exposure process: (**a**) Network structure (300 °C for 2 h); (**b**) Ripening structure (300 °C for 24 h); (**c**) Partially spherical structure (300 °C for 48 h); (**d**) Completely spherical structure (300 °C for 100 h).

The hardness change in the second stage of the 300 °C thermal exposure process is mainly associated with the microstructure evolution of the Ni-rich phases as suggested. The morphologies of Ni-rich phases have been changed during the thermal exposure, as presented in Figure 4a–d. Indeed, there are three types of Ni-rich phases morphologies during exposure process, beginning from network structure to ripening structure and finally to spherical cluster structure. For instance, the Ni-rich phases with network structure (2 h, Figure 4a) are evolved to the ripening structure (24 h, Figure 4b) when the alloy is exposed to 300 °C. Then, the Ni-rich phases with ripening structure should partially be transformed into the spherical cluster structure after the alloy is exposed to 300 °C for 48 h (Figure 4c). Finally, most Ni-rich phases are transformed into the spherical cluster structure when the thermal exposure process can last for 100 h (Figure 4d).

Compared with the initial network structure of the Ni-rich phases, these phases with ripening structure have larger roundness and coarser characteristics (Figure 4a,b). Notably, the Ni-rich phases with ripening structure can still maintain their initial interconnected morphological features. Besides, the Ni-rich phases with spherical cluster structure has completely lost of the interconnection characterization, which means the reduction of their load carrying capacity. For example, Zuo et al. reported that the reduction of the alloy’s mechanical properties was due to the morphological transformation of the Ni-rich phases, which was from the network structure to the spherical-shape during the exposure process [14].

**Figure 5:**
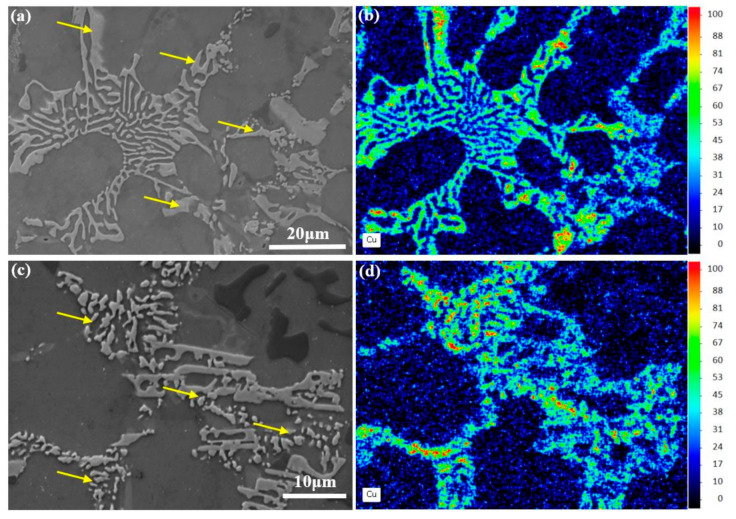
(**a**,**b**) First transition state of Ni-rich phases, from network structure to ripening structure and corresponding Cu distribution map; (**c**,**d**) Second transition state of Ni-rich phases, from ripening structure to spherical cluster structure and corresponding Cu distribution map.

Two transition states are found among these three morphologies of Ni-rich phases during 300 °C exposure process (Figure 5), which can be distinguished by differences in the morphological features and Cu content. The first transition state of the Ni-rich phases during thermal exposure process is shown in Figure 5a, which is from the network structure to the ripening structure (marked by a yellow arrow). In particular, the red parts (Figure 5b) have a higher Cu content, which are the Ni-rich phases with ripening structure. Quantitatively, the Cu/Ni ratio increases from 1.2 (network structure) to 1.5 (ripening structure). This means that the ripening process of the Ni-rich phases starts from the edge part of the Ni-rich phases during this process. Figure 5c,d show the second transition state of Ni-rich phases, which is from ripening structure to spherical cluster structure (marked by a yellow arrow). The Cu content of the Ni-rich phases with spherical cluster structure (the Cu/Ni ratio is 2.7) is higher than the ripening structure (the Cu/Ni ratio is 1.5). Thus, the existence of the composition differences among three morphologies of Ni-rich phases has been confirmed.

The micro-hardness of the Ni-rich phases with these three morphologies (network structure, ripening structure and spherical cluster structure) are 167.3 HV, 204.4 HV and 114.2 HV, accordingly. Conclusively, the Ni-rich phases with ripening structure has the largest micro-hardness, and the spherical cluster structure has the smallest micro-hardness. Namely, the micro-hardness differences are also determined among these three morphologies Ni-rich phases.

To summarize, the relationship between Cu content of these three morphologies Ni-rich phases is: network structure < ripening structure < spherical cluster structure. The Cu diffusion during thermal exposure process plays an important role in the morphological evolution of Ni-rich phases. Furthermore, the relationship between the micro-hardness of three Ni-rich phases is: spherical cluster structure < network structure < ripening structure. The appearance of the Ni-rich phases with spherical cluster structure is the main reason for the hardness decrease in the second stage of the 300 °C thermal exposure process. Generally, the Ni-rich phases with ripening structure have the obvious strengthening effect during thermal exposure process. The differences in the composition and the micro-hardness among these three morphologies for Ni-rich phases have been both confirmed. The contribution of the Ni-rich phases to the alloy hardness during thermal exposure is to be discussed in the next section.

### 4.2. Thermal Stability of T6(235 °C) Al-Si Alloy under 250–400 °C Thermal Exposures

**Figure 6:**
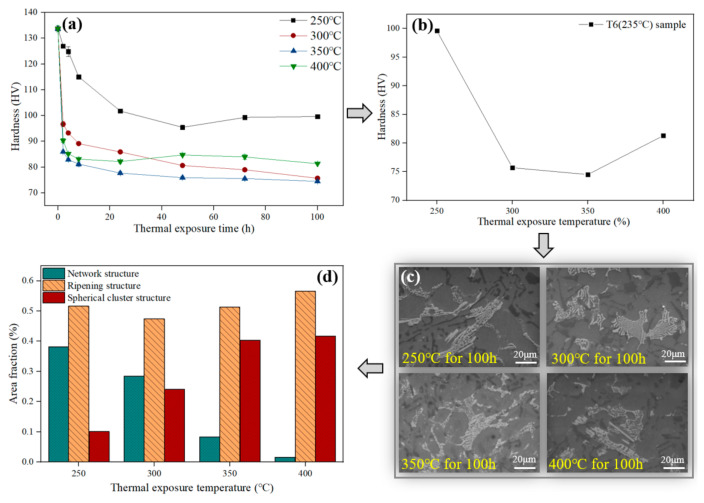
Quantitative analysis process: (**a**) Exposure curves of T6 (235 °C) Al-Si alloy; (**b**) The isochronous temperature curve (Final hardness-Temperature) of the alloy; (**c**) Microstructure of the alloy after 100 h exposure; (**d**) Quantitative statistical results of three Ni-rich phases with different morphologies.

Thermal exposure experiments at various temperatures are carried out to offer the more comprehensive understanding of the hardness changes of the Al-Si piston alloy under actual working conditions. Furthermore, the relationship between the Ni-rich phases and the alloy hardness evolution should be discussed by four main steps (Figure 6). The hardness evolution of T6 (235 °C) alloy under different exposure temperatures is displayed in Figure 6a. There are four exposure curves showing the similar hardness changing trends, where a sharp hardness decreases in the first few hours, and then slows down in subsequent exposure process. The differences are exhibited in their hardness decreasing rate in the first stage of exposure process and the final hardness of the alloy. For instance, the alloy has the smallest hardness decreased rate (1.3 HV/h) and the largest final hardness (103.5 HV) during 250 °C exposure process. Conversely, the maximum hardness reduction rate (26.5 HV/h) and the minimum final hardness (79.3 HV) have found in the 350 °C thermal exposure process instead of 400 °C.

The alloy’s thermal stability can be judged by the final hardness. Figure 6b shows the isochronous temperature curve, which illustrates the relationship between the final hardness and the exposure temperature for the alloy [31]. For instance, the final hardness shows a downward trend when the temperature is in the range of 250 °C to 350 °C. Comparatively, the final hardness of the alloy shows an upward trend, which increases by 6.8 HV when the temperature rises from 350 °C to 400 °C. Since the morphology evolution of the second phase is closely related to the alloy hardness, Figure 6c shows the morphologies of these four alloys under different exposed states. Morphological evolutions of the Si phase are similar in these four states, i.e., the roundness of primary Si phase and the disconnection and spheroidization of eutectic Si phase. Interestingly, Ni-rich phases with spherical structure also appeared in the alloy which was exposed at 250 °C for 100 h. Thus, these three types of Ni-rich phases have appeared in four alloys simultaneously. Quantitative analysis should be used in the fourth step to illustrate the influence of the high temperature on the morphology of Ni-rich phases.

The statistical results are shown in Figure 6d based on the Ni-rich phases classified in Figure 4. The histogram results indicate that these three Ni-rich phases of the alloy exposed at different temperatures occupied different area fractions. For instance, the area fraction of Ni-rich phases with the network structure and spherical structure are both linearly related to exposure temperatures. Moreover, the former shows a downward trend while the latter shows an upward trend with the increasing exposure temperature. Secondly, the area fraction of the Ni-rich phases with ripening structure has a U-shaped relationship with the increasing exposure temperatures (from 250 °C to 400 °C). Conclusively, the area fraction of the Ni-rich phases with ripening structure decreases when the temperature ranges from 250 °C to 350 °C, and then increases when the temperature goes from 350 °C to 400 °C.

It is universally accepted that the Ni-rich phases can act as the main heat-resistant phase during the exposure process [9]. Notably, the morphology and the micro-hardness of the Ni-rich phases have changed significantly by the thermal exposure. Therefore, it is necessary to find a quantitative method to illustrate the hardness change of the Ni-rich phases after different temperatures exposure.

Based on the test principle of the Vickers hardness tester, the hardness value of the alloy is closely linked with the type of the second phases [29]. Consequently, the weighted micro-hardness (*HV_N_*) of the Ni-rich phases can be used to illustrated the actual hardness evolution of Ni-rich phases. The calculation formula of weighted hardness of Ni-rich phases is as follows:

(2) $$HV_{N}=\sum_{i=1}^{3}\left(HV_{i}\cdot f_{i}\right)$$

where *HV_N_* is weighted micro-hardness of Ni-rich phases; *f_i_* is the area fraction of the *i*-type Ni-rich phases; *HV_i_* is the micro-hardness of the *i*-type Ni-rich phases. (Notes: Type 1 is the Ni-rich phases with network structure; Type 2 is the Ni-rich phases with ripening structure; Type 3 is the Ni-rich phases with spherical cluster structure.)

The weighted micro-hardness values of Ni-rich phases are presented in the last column data in Table 3. It is similar to the changing trend presented in Figure 6b. With the increasing exposure temperature, the weighted micro-hardness of the Ni-rich phases decreases first (from 250 °C to 350 °C), and then increases slightly (from 350 °C to 400 °C). Accordingly, the *HV_N_* of the Ni-rich phases should be discussed in both temperature ranges.

The difference in weighted hardness can be explained from the morphological evolution of the Ni-rich phases. In the first temperature range (from 250 °C to 350 °C), the weighted micro-hardness *HV_N_* of the Ni-rich phases drops from 181.0 HV to 172.1 HV, and finally 164.3 HV. The area fraction of Ni-rich phases with spherical cluster structure (*f_3_*) is doubled for every 50 °C increase in the temperature (i.e., 250 °C/10.1%, 300 °C/24.1%, 350 °C/40.4%). Meanwhile, the area fraction of Ni-rich phases with network structure (*f_1_*) decreases significantly in this temperature range. Notably, the area fraction of the Ni-rich phases with ripening structure (*f_2_*) is kept at a relative stable level (i.e., 250 °C/51.5%, 300 °C/47.4%, 350 °C/51.3%). This suggests that the decreased trend is associated with the increasing *f_3_* and the decreasing *f_1._* On the other hand, *HV_N_* is increased by 1.8 HV when the temperature rises from 350 °C to 400 °C. The improvement of *HV_N_* can be attribute to the 5.3% increase of *f_2_*, which can offset the hardness reduction effect of 1.4% increase of *f_3_*. Meanwhile, the area fraction of Ni-rich phases with network structure decreases from 8.3% to 1.5%.

Based on the analysis of *HV_N_*, Ni-rich phases with the ripening structure plays an important role in improving the thermal stability of the alloy. On the contrary, the formation of Ni-rich phases with spherical cluster structure is the main cause of the degradation in thermal stability for the alloy.

### 4.3. The Effect of Thermal Exposure on the Micro-Hardness of Al Matrix

**Figure 7:**
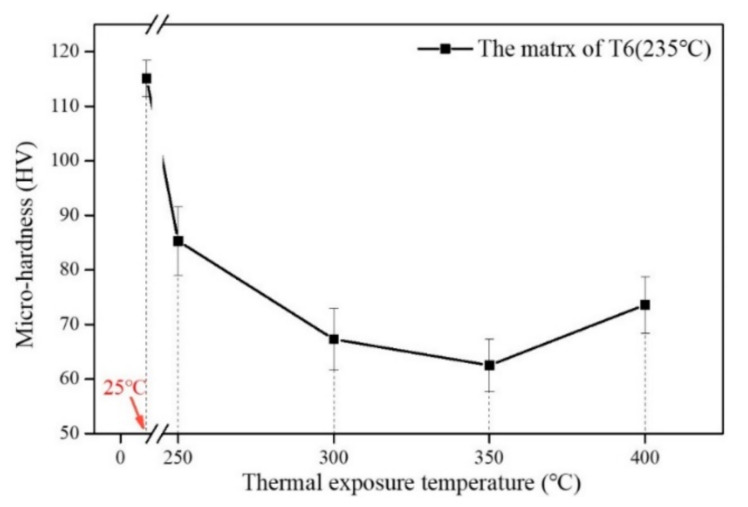
Isochronous temperature curve (The final micro-hardness value of matrix-Temperature) of T6 (235 °C) Al-Si alloy.

In addition to the Ni-rich phases, the matrix also plays an important role in the alloy thermal stability. Generally, the strength of the Al matrix is mainly from the precipitation effect [43]. Therefore, it is necessary to explore the relationship between the precipitates and the alloy hardness under different exposure temperatures. The micro-hardness change trend of the matrix with exposure temperatures is showed in Figure 7. The micro-hardness of the matrix is decreased from 114.5 HV to 85.3 HV once the T6 (235 °C) alloy is exposed to 250 °C for 100 h. Reported by Sui et al., this was mainly due to the coarsening of precipitate to decrease its precipitation strengthening effect [44]. The micro-hardness of the matrix is further reduced to 67.3 HV (300 °C for 100 h) and 62.5 HV (350 °C for 100 h). As stated by Liu et al. [45], the mechanical properties of the Al-Si alloy are improved by the θ-Al_2_Cu precipitate below 250 °C. However, its performance drops sharply at 300 °C due to the higher diffusion rate and solubility of Cu in the matrix at higher temperature. The morphological evolutions of the precipitates in the Al-Si alloy for both 300 °C and 350 °C exposure processes were reported by Liu et al. [46] and Tian et al. [16]. The precipitation was rarely changed after the precipitates rapidly coarsening during the first few hours of exposure process. Moreover, the higher temperature provides the greater driving force for the precipitates coarsening [47].

The final micro-hardness of the matrix is increased by 11.1 HV when the temperature rises from 350 °C to 400 °C. The upward trend is similar to the hardness evolution (Figure 6b) and the weighted micro-hardness evolution of the Ni-rich phases (Table 3). Normally, the increase in the micro-hardness of the matrix can be attributed to the formation of some novel dispersoids in this complicated alloy [48].

**Figure 8:**
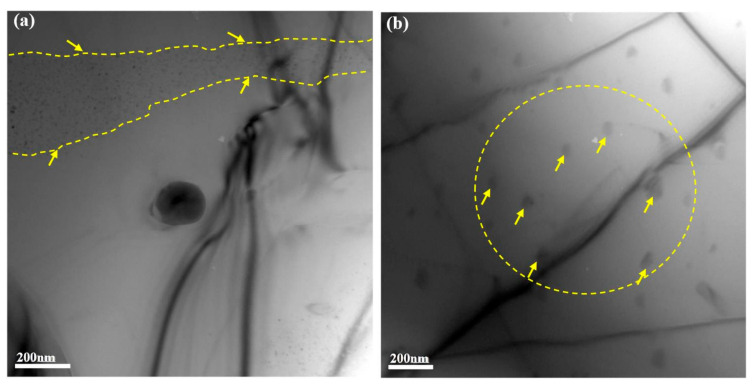
TEM observations of precipitate in matrix (exposed to 400 °C with 100 h duration): (**a**) The precipitate with smaller size and denser distribution in the matrix; (**b**) The precipitate with larger size and more dispersed distribution in the matrix.

Figure 8a,b show the dispersoids morphologies of the Al-Si alloy after exposed at 400 °C for 100 h, and two different sizes of the precipitates are characterized. The smaller size (Figure 8a, about a few nanometers) dispersoids with approximately spherical shape are spread in the matrix. The larger size (Figure 8b, over one hundred nanometers) dispersoids with irregular block shape evenly distributed in the matrix. Besides, the black lines are dislocation lines or stress lines, which may be induced during sample preparation process.

The TEM-EDS results of the dispersoids inside the yellow dotted line (Figure 8) were showed in Table 4. Two dispersoids of different sizes shared the similar compositions (i.e., Al, Cu and Fe). The atomic ratio of Cu and Fe is 1:1 from the experimental data. Besides, the sum atomic concentration of Cu and Fe is about half of the Al atoms. It can be inferred that Fe atoms replaced half of Cu atoms in the θ-Al_2_Cu dispersoid. Therefore, the dispersoid can be regarded as Al_2_(Cu, Fe), which act as an important factor for improving the micro-hardness of the matrix in this condition.

Similarly, some new dispersoids have also been reported at elevated temperature in the available references [16,46,49]. Sui et al. found that the addition of Gd induces Al_3_CuGd dispersoid formation at grain boundaries, which can simultaneously improve the strength of the eutectic Al-Si alloy at both room and elevated temperature [49]. According to Tian et al., the Al_11_Cu_5_Mn_3_ dispersoid was formed after re-aging at 420 °C, which has an obvious precipitation strengthening effect in the Al-Si alloy. Therefore, both the tensile strengths (both at room temperature and 350 °C) and the high cycle fatigue life of the alloy were improved after the alloy re-aged at 420 °C [16]. Therefore, many different dispersoids have been reported in the complex Al-Si alloys at elevated temperature, and have yet been made any consensus neither in structural nor compositional information for the dispersoid. For this reason, the further research is required to explore the key microstructure features for the improvement on the alloy hardness at higher temperatures of 400 °C.

## 5. Conclusions

In this study, the effect of thermal exposure (250–400 °C) on the hardness of the Al-Si piston alloy has been analyzed based on the microstructure evolution during this process. Our work has systematically described and explained the Al-Si alloys thermal stability from the relationship between microstructure feature-mechanical property (hardness). Based on the previous work, we can draw the following conclusions:

(1) Through the T6 treatment, the lower aging temperature can induce the higher hardness among four PA states (160 °C, 180 °C, 200 °C and 235 °C). However, the final hardness values are similar in these states by the thermal exposure. Normally, based on the hardness evolution of the alloys and micro-hardness fluctuation of matrix, the thermal exposure process can be divided into two stages using T6 (235 °C) as an example. The first stage is associated with coarsening of the precipitates, and the second stage is correlated with the evolution of the Ni-rich phases.
(2) The morphological evolution of the Ni-rich phases during the thermal exposure has followed the sequence: network structure → ripening structure → spherical cluster structure. The Cu content sequence is: network structure < ripening structure < spherical cluster structure. The micro-hardness sequence is: spherical cluster structure < network structure < ripening structure. The Ni-rich phases with ripening structure has played an important role in improving the thermal stability of the alloy. The simultaneous decrease of the network structure and the increase of the spherical cluster structure should cause the hardness reduction of the alloy.
(3) Statistical results of the Ni-rich phases show that the area fractions of three morphologies have varied with the exposure temperature. The network phase and spherical cluster phase change linearly with the increasing exposure temperature, and the former is inversely proportional while the latter is in direct proportion. However, the area fraction of the Ni-rich phases with ripening structure changes with exposure temperature in a U-shaped curve.
(4) The hardness of the Al-Si alloy, the micro-hardness of the matrix and the weighted hardness of the Ni-rich phases have obeyed the similar trend with exposure temperature. Their values decrease in the temperature range of 250 °C to 350 °C, and then increase when the temperature climates to 400 °C.
(5) The hardness improvement at 400 °C can be attributed to both the increase of the area fraction of Ni-rich phases with ripening structure and the precipitation of new dispersoids Al_2_(Cu, Fe).

## Figures and Tables

**Table 1:** Chemical composition of alloy (wt.%) characterized by ICP-AES.

**Element**	Si	Cu	Ni	Mg	Mn	Fe	Al
**Nominal Composition**	12.00	4.00	2.00	1.00	0.10	0.10	Bal
**ICP Value**	12.12	4.00	1.86	0.94	0.10	0.10	Bal

**Table 2:** Hardness details of the Al-Si alloy at four PA states.

	Peak Aged Hardness (HV)	Final Hardness (HV)	ΔHV (HV)	Hardness Retention Ratio
T6 (160 °C)	151.1 ± 0.3	77.1 ± 0.5	−75.4	50.6%
T6 (180 °C)	149.7 ± 1.5	75.5 ± 0.7	−69.3	52.1%
T6 (200 °C)	145.7 ± 1.6	75.9 ± 0.7	−71.8	51.4%
T6 (235 °C)	136.0 ± 1.0	75.7 ± 0.4	−58.0	56.6%

**Table 3:** Area fraction and weighted hardness of T6 (235 °C) Al-Si alloy after 100 h exposure at different temperatures.

	*f_1_* (%)	*f_2_* (%)	*f_3_* (%)	*HV_N_* (HV)
250 °C	38.3	51.5	10.1	181.0
300 °C	28.4	47.4	24.1	172.1
350 °C	8.3	51.3	40.4	164.3
400 °C	1.5	56.6	41.8	166.1

**Table 4:** TEM-EDS results of dispersoids.

	Atomic Percentage (%)	
	(a)	(b)
Al	69.7	67.8
Cu	15.6	16.3
Fe	14.7	15.9
